# Emergence of new *Salmonella *Enteritidis phage types in Europe? Surveillance of infections in returning travellers

**DOI:** 10.1186/1741-7015-2-32

**Published:** 2004-09-02

**Authors:** Karin Nygård, Birgitta de Jong, Philippe J Guerin, Yvonne Andersson, Agneta Olsson, Johan Giesecke

**Affiliations:** 1 European Programme of Intervention Epidemiology Training (EPIET); 2 Department of Infectious Disease Epidemiology, Swedish Institute for Infectious Disease Control, 171 82 Solna, Sweden; 3 Department of Infectious Disease Epidemiology, Norwegian Institute of Public Health, 0403 Oslo, Norway

## Abstract

**Background:**

Among human *Salmonella *Enteritidis infections, phage type 4 has been the dominant phage type in most countries in Western Europe during the last years. This is reflected in *Salmonella *infections among Swedish travellers returning from abroad. However, there are differences in phage type distribution between the countries, and this has also changed over time.

**Methods:**

We used data from the Swedish infectious disease register and the national reference laboratory to describe phage type distribution of *Salmonella *Enteritidis infections in Swedish travellers from 1997 to 2002, and have compared this with national studies conducted in the countries visited.

**Results:**

Infections among Swedish travellers correlate well with national studies conducted in the countries visited. In 2001 a change in phage type distribution in *S. *Enteritidis infections among Swedish travellers returning from some countries in southern Europe was observed, and a previously rare phage type (PT 14b) became one of the most commonly diagnosed that year, continuing into 2002 and 2003.

**Conclusions:**

Surveillance of infections among returning travellers can be helpful in detecting emerging infections and outbreaks in tourist destinations. The information needs to be communicated rapidly to all affected countries in order to expedite the implementation of appropriate investigations and preventive measures.

## Background

*Salmonella *Enteritidis is the most common serovar causing food-borne salmonellosis in humans, causing approximately 80% of salmonellosis cases reported in Europe [1]. During the 80s and early 90s, a steady increase in *S. *Enteritidis infections was reported in Europe and North America [2-4]. The most common phage types of *S*. Enteritidis varies between countries; while phage type (PT) 4 is reported to be dominant in most countries in Western Europe, PT 8 is common in North America and also a few European countries [5-7]. Epidemiological and environmental studies have implicated eggs and poultry products as primary risk factors for infection [3,8]. Approximately 70% of outbreaks caused by S. Enteritidis in Europe during the 90s, were related to eggs and egg products [1]. Based on these findings, prevention and control measures in the egg and poultry industry have been implemented in the European Union and in the US [9,10]. These measures seem to have been effective in reducing *S*. Enteritidis contamination of eggs [11] and are believed to have lead to a decrease in human incidence of *S. *Enteritidis in recent years [1,9,12].

In Sweden, about 85% of the reported salmonella infections are acquired during travel abroad and the levels in domestic animals and food products is low [13]. Therefore, trends in human salmonellosis in Sweden have mainly reflected trends in foreign travel and countries with popular package tour resorts account for the majority of infections.

In this study, we investigate trends in travel related *S. *Enteritidis infections, describe the phage type distribution of *S. *Enteritidis isolated from Swedish travellers returning from abroad related to country of infection, and discuss possible reasons for the emergence of a new phage type of *S. *Enteritidis in 2001. A preliminary analysis of a subset of these data was published as a short letter in the *Lancet *in 2002 [14].

## Methods

In Sweden, salmonellosis is a mandatory notifiable disease. Both clinicians and laboratories are required to report a case to the infectious disease register at the Swedish Institute for Infectious Disease Control (SMI). Based on the patient's travel history, information on probable country of infection is collected on the notification forms. Diagnosis of salmonella infections is made at regional microbiology laboratories, and all isolates are submitted to the national reference laboratory at SMI for serotyping and phage typing. In this study we have included all cases of *S.*Enteritidis notified to SMI from January 1, 1997 through December 31, 2002.

To investigate trends in travel-related infections, we collected information on air travel from the Swedish Civil Aviation Administration (CAA)[15]. The figures include all passengers carried on flights from any CAA airport to their first foreign destination, without indicating whether this destination is for transfer or the final destination. Using these figures as denominators, we calculated annual incidence rates for countries reported as place of infection for the three most popular countries for charter tourism among Swedes – Spain, Greece and Turkey. The incidence rates were calculated by dividing the number of cases reported as infected in the respective countries by number of flight passengers to that country.

For the geographical description of dominant phage types in the different countries the analysis was restricted to infections acquired in countries in Europe during 1997 to 2002, and to countries from which more than ten cases were reported during this period. A phage type was considered dominant if it represented >30% of the isolates and was at least twice as common as the second most common phage type. If none of the phage types fulfilled these criteria, the two most common phage types that together represented >50% of the cases were defined as the dominant types. The pattern for 1997 to 2000 was compared with the pattern for 2001.

## Results

Of 13,271 cases of *S.*Enteritidis infections notified during 1997–2002, 11,570 cases (87%) were reported as infected abroad, 1,032 (8%) were reported as infected in Sweden, and information of probable country of infection was not available for 669 (5 %). The total number of cases reported each year varied from 1,598 to 2,629 cases, with the highest being reported in 1999 and the lowest in 2002. Imported cases varied between 1,404 and 2,164. The most common countries of infection during the six-year period were Spain, Greece and Turkey, accounting for 34%, 8% and 4% of all cases, respectively.

**Figure 1:**
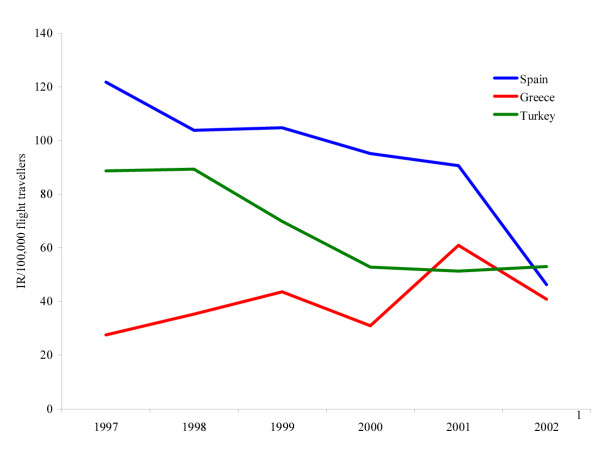
Incidence rates (IR) of *Salmonella *Enteritidis infections among Swedish travellers to Spain, Greece and Turkey. Country specific annual incidence rate of *Salmonella *Enteritidis per 100,000 air travellers from Sweden. Incidence rates are calculated based on the number of cases notified to the Swedish infectious Disease register using the number of flight passengers from Sweden to the first foreign destination as the denominator.

For six countries – 'Spain, Greece, Turkey, Poland, Thailand and Portugal – >50 cases of infection with *S*. Enteritidis among Swedish travellers were reported each year during 1997 to 2002. These are all popular destinations for leisure travel among Swedes. Figure 1 presents incidence rates for the three most popular countries for charter tourism, Spain, Greece and Turkey, using the number of flight passengers from Sweden to the first foreign destination as the denominator. The figure shows that the incidence rate among travellers to Spain and Turkey seemed to decrease during the six-year period, while for Greece the incidence peaked in 2001.

**Figure 2:**
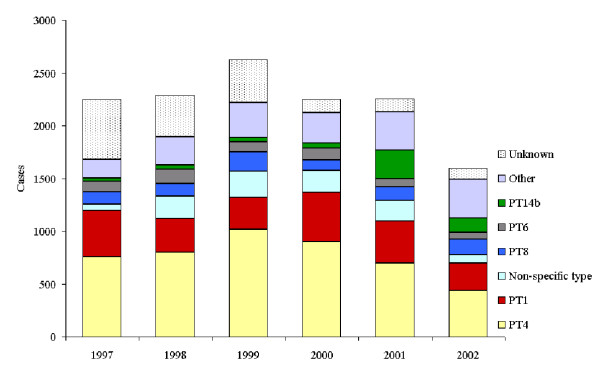
Distribution of phage types of *Salmonella *Enteritidis cases notified in Sweden, 1997 to 2002.

Eighty-six percent (10,049) of the isolates from 1997 to 2001 were phage-typed, increasing from 75% in 1997 to 95% in 2001. The most common phage types over the period were PT 4 and PT 1 (Figure 2), accounting for 35% and 16% of all cases, respectively. In travellers returning from most countries in Western Europe, PT 4 was the dominant phage type. In Eastern Europe, PT 1 was dominant, and this phage type was also common among travellers returning from the Iberian Peninsula. PT 8 seemed to be more common among travellers returning from central European countries.

**Figure 3:**
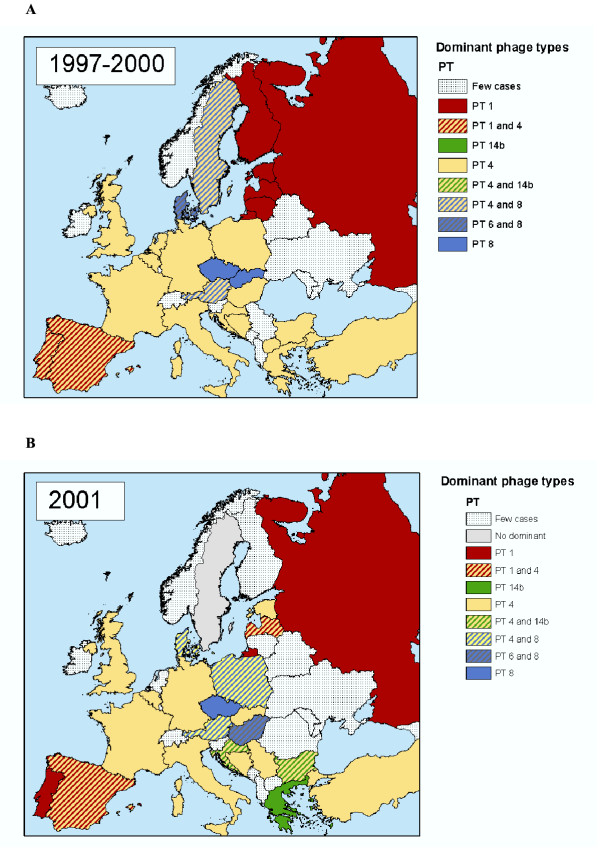
Dominant phage types of *Salmonella *Enteritidis infections among Swedish travellers according to country visited. Only countries with more than 10 reported cases and phage types accounting for > 25 % of all cases are shown. a) 1997 – 2000 b) 2001

**Figure 4:**
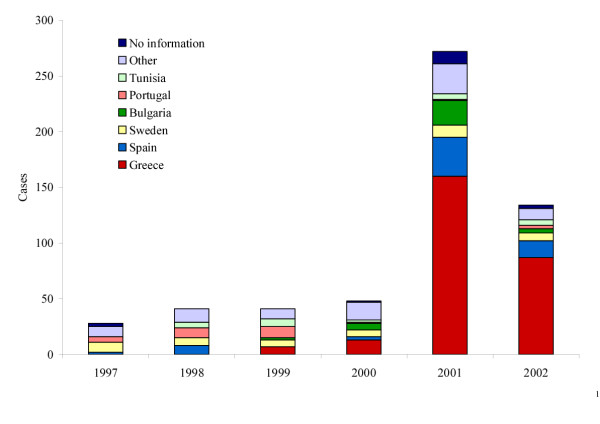
Notified cases of *Salmonella *Enteritidis PT 14b among Swedish travellers by country of infection, 1997 – 2002.

In 2001 this pattern changed when PT 14b increased among travellers from several countries in Southern Europe (Figure 3b). PT 14b was the third most common phage type among returning travellers in 2001, accounting for 13% of all isolates that were phage-typed that year (272/2,132), compared with 2 % of all that were phage-typed in the previous four years (154/7,917). This trend continued into 2002 when PT14b accounted for 9% (134/1,489) of all typed isolates. The majority of the PT 14b cases in 2001 and 2002 were reported among travellers returning from Greece (Figure 4), and this phage type accounted for 54%(157/293), 47%(83/176) and 42%(50/117) of all cases of *S. *Enteritidis from Greece in 2001, 2002 and 2003, respectively.

## Discussion

We have described trends and phage type distribution of *S. *Enteritidis isolates among Swedish travellers infected abroad. Phage type 4 was the dominant phage type among returning travellers. There were, however, some differences in distribution between the countries and with time. Between 1997 and 2000, PT 1 dominated among travellers returning from Russia and the Baltic countries, PT 8 was commonly seen among travellers returning from some central European countries, and PT 4 dominated among travellers returning from most other European countries (Figure 3). In 2001 a change in phage type distribution was observed among Swedish travellers returning from some countries in South-Eastern Europe. PT 14b, a previously rare phage type, appeared to become predominant among travellers returning from Greece and also became more common among travellers from some other countries.

During the six-year period, the *S*. Enteritidis incidence rate among travellers to Spain and Turkey appeared to decline. This trend is in contrast to surveillance data from Spain, which seem to show an increasing incidence over the same period [16]. The reason for this declining trend among tourists was not investigated, but may be related to an increased awareness among tourists concerning the prevention of traveller's diarrhoea or to improved food control efforts in some of the popular tourist resorts.

There are some uncertainties when calculating incidence rates based on number of travellers. The number of travellers used as the denominator in the incidence rate calculations is based on the statistics of the airport of first landing after leaving Sweden. If the first airport is not the final destination, these travellers will not be included in the denominator for the destination country. Thus the calculated incidence rates for transit countries will be too low, while the incidence rates for the final destination countries will be too high.

When comparing *S. *Enteritidis phage types isolated from Swedish travellers with studies conducted in the countries visited, isolates found in travellers were generally consistent with the dominant strains reported among inhabitants in the respective countries. Table 1 summarises the results of published studies on salmonellosis from a number of countries. The percentages of PT 4 and PT 6 among human *S*. Enteritidis infections in Austria and Denmark, respectively, have been reported to be decreasing in the last years [6]. These same trends were reflected among returning Swedish travellers.

People returning from travel abroad may have a higher tendency to seek medical care and have a stool sample taken if an imported infection is suspected. In addition, visitors may be more susceptible to pathogens circulating in the community than the local inhabitants. The detection of new, emerging strains in travellers after returning to their home countries may therefore be helpful in detecting changes in the pathogen reservoir occurring in the countries visited, especially in tourist destinations. However, tourists have a tendency to aggregate in some smaller resorts that may have a different pathogen reservoir and rely on food supplies that are different from the rest of the country. This may lead to differences in risks and pathogens between the inhabitants and the tourists visiting the country that needs to be taken into consideration.

The change in phage type distribution observed among Swedish travellers returning from some countries in Southern Europe in 2001 was not observed among inhabitants in the countries visited. In total, the two most common phage types among Swedish travellers were, as in previous years, PT 4 and PT 1. However, the third most common was PT 14b, a phage type hitherto uncommon in Sweden with only 20 to 40 cases reported annually prior to 2001. The majority of the cases were among travellers returning from Greece (90%). More cases were also reported among travellers returning from Spain and Bulgaria than in previous years. During the same time period, an increase in the same phage type had also been registered among Norwegian and Finnish travellers returning from Greece [17]. A request on Enter-Net (European network for the surveillance of enteric infections – *Salmonella *and VTEC O157) sent by the Norwegian Public Health Institute gave no response on increases of PT 14b in other countries. Spain reported an outbreak of the same phage in a school in January (unpublished data). But after this event, no further increase was noted. The UK later reported an increased number of the same phage type, both among travellers and among people who had been infected in the UK. However, there the 14b isolates of domestic origin were aerogenic, while isolates associated with travel to Greece were predominantly anaerogenic [18]. The isolates among Swedish travellers returning from Greece were also predominantly anaerogenic.

No explanation for the sudden increase in this phage type among Nordic travellers to different countries in Southern Europe has been found. PT 14b is not a new phage type and outbreaks reported previously have been mainly related to eggs and egg-products (ice-cream, tiramisu [19,20]) or improper hygiene practices [21,22]. However, these outbreaks were localised, of limited duration, and the incriminated food products found and the outbreaks contained. The cause of the increase of this phage type among Nordic travellers in 2001 is still unclear. It may represent a geographically more widespread outbreak than previously described, possibly due to increased trade in food products, animals or animal feed across the borders. Another possible explanation for this increase may be that changes in PT 14b could have contributed to increased resistance or virulence factors, thereby facilitating the spread of this phage type in the environment. Alternatively, acquisition or loss of a plasmid or spontaneous mutations may have resulted in a conversion from another phage type to PT 14b. Such change has been described for other phage types [23-25] and a conversion from PT 8 to PT 14b has been described after inoculation into pathogen-free chicken [26].

Our data presented are limited by the small numbers of cases from each country investigated on an annual basis. It is therefore difficult to evaluate trends with any certainty. However, the possibility of using surveillance data of infections among returning travellers to detect emerging pathogens should be further investigated. In addition, data from countries that routinely collect information on travel could be pooled in order to increase the numbers of travel-related infections. In several countries outside Europe, laboratory capacity is limited and it may take a long time to detect the emergence of new pathogens or subtypes. Not all countries in Europe collect information on the probable place of infection and phage typing of *Salmonella *isolates is not routinely performed in some countries. However, if available, this information will be included in the data reported to Enter-Net. Data from this network has previously been useful in detecting travel-related outbreaks [27,28], and may also be useful in describing pathogen patterns in countries where laboratory capacities are limited or routine typing is not performed. Importantly, Enter-Net may expedite the dissemination of information concerning emerging pathogens.

## Conclusions

This study demonstrates that surveillance of infections among returning travellers may be helpful in detecting emerging infections and outbreaks in tourist destinations, and provides some useful supplementary data about infectious diseases and trends in other geographical regions. Characterization of isolates from travellers can detect changes in the pathogen and antimicrobial resistance patterns in the destination country. This information may be an important supplement in countries where surveillance systems are deficient or lacking, or where the laboratories have limited capacity to do detailed sub-typing and resistance testing. In addition, infections and outbreaks among tourists may not always affect the local residents and therefore may not be detected by the local public health authorities. If proper investigations, and appropriate prevention and control measures are to be implemented in the countries visited, it is important that the surveillance information compiled from the traveller's home countries is rapidly communicated to the affected countries.

## Competing interests

None declared.

## Authors' contributions

KN performed the data analysis and drafted the manuscript. PJG, YA and BdJ participated in the design and coordination of the study. AO conducted typing and provided advice regarding laboratory issues. JG participated in the design and discussion, and provided advice on data analysis. All authors read and approved the final manuscript.

## Pre-publication history

The pre-publication history for this paper can be accessed here:



## Figures and Tables

**Table 1:** Dominant Salmonella Enteritidis phage types by country*. Comparison between findings among Swedish travellers and studies conducted in the countries visited.

Country	Number of *S*. Enteritidis-infections notified among Swedish travellers 1997–2001	Most common *S*. Enteritidis phage types among returning Swedish travellers 1997–2001(%*)	Predominant phage type described in studies from the country visited	Year of study	Reference
Spain	4125	1 (33%), 4 (36%)	1, 4, 6	1990s	[29]
Greece 1997–2000	916	4 (44%) 14b (59%)			
Turkey	496	4 (65 %)	4	Not mentioned	[30]
Poland	354	4 (40 %)	4, 8	1986–95	[31]
Portugal	344	1 (43 %)			
Morocco	310	4 (50 %)			
Germany	235	4 (59%)	4	1998	[32]
Denmark 1997–1998 1999 2000–2001	223	6 (42%), 8 (43%) 34 (73%) 4 (30%), 8(30%)	6, 8 PT34 outbreak in DK 4, 8	1997–98 1999 2000–2001	[33]
Czech Republic	194	8 (68%)	8	1989–98	[34]
Bulgaria	180	4 (55%			
Cyprus	166	1 (36%), 4 (45%)			
Tunisia	152	4 (75%)			
Italy	152	4 (47%)	4	1990–93	[29,35]
United Kingdom	133	4 (76%)	4	1997–2000	[36]
Hungary	120	4 (50%)	4	1992–94	[37]
France	110	4 (56%)			
Austria 1997–2000 2001	81	4 (52%), 8 (38%) 4 (33%), 8 (42%)	4	1995–2001	[6]
Latvia 1997–2000 2001	76	1 (63%) 1 (60%), 4 (40%)			
Egypt	63	4 (55%)			
Croatia	53	4 (63%)			
Belgium	45	4 (72%)			
Russia	42	1 (62%)	1	1980–93	[38]
Estonia 1997–2000 2001	37	1 (58%) 1 (25%), 4(75%)**			
Bosnia and Herzegovina	26	4 (64%)			
The Netherlands	20	4 (75%)	4	1997–2001	[6]
Slovakia	14	8 (62%)	8	1995	[39]
Lithuania	12	1 (50%)			

* Calculated as a percentage of all isolates that were phage-typed during 1997–2001.

** Based on less than 10 cases.
